# Complement System and Potential Therapeutics in Age-Related Macular Degeneration

**DOI:** 10.3390/ijms22136851

**Published:** 2021-06-25

**Authors:** Young Gun Park, Yong Soo Park, In-Beom Kim

**Affiliations:** 1 Department of Ophthalmology and Visual Science, Seoul St. Mary’s Hospital, College of Medicine, The Catholic University of Korea, Seoul 06591, Korea; cuteyg2000@catholic.ac.kr; 2 Department of Anatomy, College of Medicine, The Catholic University of Korea, Seoul 06591, Korea; yongsoopark88@gmail.com; 3 Catholic Neuroscience Institute, College of Medicine, The Catholic University of Korea, Seoul 06591, Korea; 4 Catholic Institute for Applied Anatomy, College of Medicine, The Catholic University of Korea, Seoul 06591, Korea

**Keywords:** age-related macular degeneration, complement cascade, clinical trial, therapeutics

## Abstract

Age-related macular degeneration (AMD) is a complex multifactorial disease characterized in its late form by neovascularization (wet type) or geographic atrophy of the retinal pigment epithelium cell layer (dry type). The complement system is an intrinsic component of innate immunity. There has been growing evidence that the complement system plays an integral role in maintaining immune surveillance and homeostasis in AMD. Based on the association between the genotypes of complement variants and AMD occurrence and the presence of complement in drusen from AMD patients, the complement system has become a therapeutic target for AMD. However, the mechanism of complement disease propagation in AMD has not been fully understood. This concise review focuses on an overall understanding of the role of the complement system in AMD and its ongoing clinical trials. It provides further insights into a strategy for the treatment of AMD targeting the complement system.

## 1. Introduction

Age-related macular degeneration (AMD) is a leading cause of vision loss in elderly people in developed countries [1,2,3]. It is related to various risk factors and genetic predispositions for a number of patients [4]. In 2020, the AMD prevalence rate was 196 million, and this is projected to increase to up to 288 million in 2040 [5]. Irreversible central vision loss has devastating effects on the physical, social, and emotional aspects of the patients and leads to a high societal cost burden.

However, the treatment options targeting vascular leakage are available only for patients with the neovascular form of the disease. Importantly, no precise curative treatment modality has been developed despite treatment advances for this disease. The most important aspects are modifiable risk factors and routine ophthalmic monitoring. This limitation of effective therapeutic options is also likely due to incomplete knowledge of the mechanisms involved in AMD pathogenesis. 

There is a lack of updated and concise papers focused on the complement system cascades associated with the pathogenesis of AMD, although there have been much research and many review articles [6,7,8,9]. In this review, we aim to provide a concise mini-review on how these pathways are affected in AMD progression and the key factors for the potential therapeutics in AMD.

## 2. Clinical Classification of AMD

**Figure 1:**
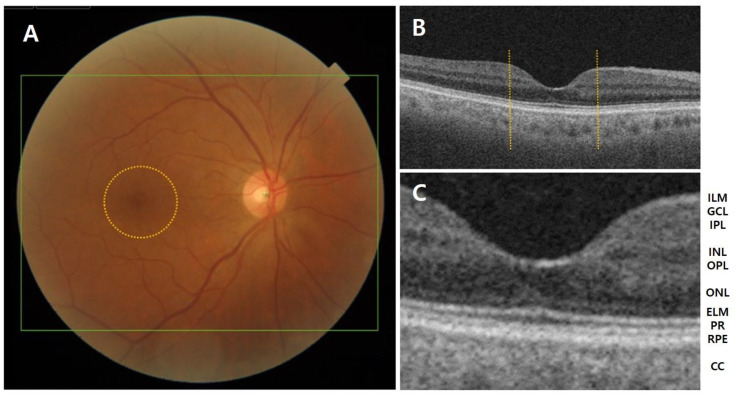
(**A**) Anatomy of the fundus and macula (circle) in a normal eye. (**B**,**C**) Layer-by-layer B-scan swept-source optical coherence tomography display of normal retina. CC, choriocapillaris; ELM, external limiting membrane; GCL, ganglion cell layer; ILM, inner limiting membrane; INL, inner nuclear layer; IPL, inner plexiform layer; NFL, nerve fiber layer; ONL, outer nuclear layer; OPL, outer plexiform layer; PR, photoreceptor; RPE, retinal pigment epithelium.

The center of the retina is the macula, and it can be divided into six parts: the umbo, foveolar, foveal avascular zone, fovea, parafovea, and perifoveal areas. Among them, the fovea is a tiny pit which is located at the center of the macula and contains the largest concentrations of cones, which are responsible for central vision with high resolution. The retina is composed of ten layers, from the anterior to posterior: inner limiting membrane, nerve fiber layer, ganglion cell layer, inner plexiform layer, inner nuclear layer, outer plexiform layer, outer nuclear layer, external limiting membrane, rods and cones (photoreceptor) and retinal pigment epithelium (RPE). Bruch’s membrane (BM) is attached to the basal surface of the RPE, and the choriocapillaris is adjacent to BM (Figure 1). However, close to the foveal pit, the layers between the ganglion cell layer and inner nuclear layer abruptly become thinner and finally disappear, while the layers between the outer plexiform layer and RPE remain, and thus, fovea is composed of a few layers, including a very thin inner plexiform layer, the outer nuclear layer, the cones, and the RPE layers.

AMD is a degenerative disease of the retina that leads to changes in photoreceptors, RPE, BM, and/or choriocapillaris, eventually resulting in central visual impairment [10]. The pathology of AMD is characterized by macular drusen, RPE atrophy, choroidal neovascularization (CNV), neurosensory retinal detachment, and disciform scars or lesions. The hallmark of AMD is drusen, extracellular debris that accumulates with age, in the area below the RPE, and consists of cellular debris, lipoproteins, and amyloid deposits. This may lead to impaired RPE function and disruption of the metabolic transport between RPE and choroid [11].

Simplified classification scales according to the clinical features for AMD have been developed. The Age-related Eye Disease Study (AREDS) research group classified patients into four categories according to the size and extent of drusen, presence of geographic atrophy (GA), and neovascular changes [12,13]. Currently, simplified classification scales according to the clinical features for AMD have been developed and have become widely used (Table 1) [14].

**Figure 2:**
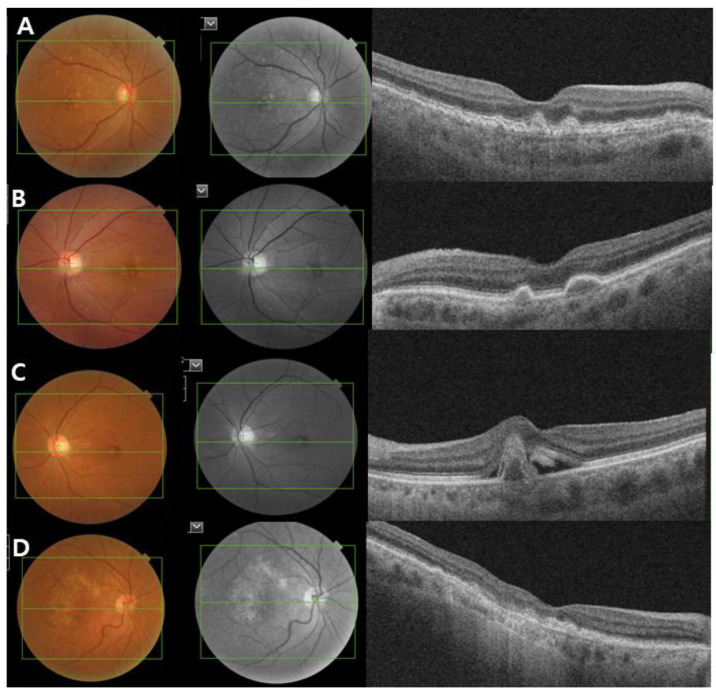
Clinical image of age-related macular degeneration (AMD). Color fundus photograph, red-free fundus photograph and swept-source optical coherence tomography (SS-OCT) images showing the characteristics of early and intermediate AMD (**A**,**B**), neovascular AMD (**C**) and geographic atrophy (**D**). (**A**,**B**) Non-neovascular AMD: Images showing small and intermediate soft drusen. (**C**) Neovascular AMD: subretinal fluid with subfoveal hemorrhage and a large pigment epithelial detachment. (**D**) Geographic atrophy: a well-demarcated area of fovea-involving retinal pigment epithelium atrophy.

AMD is categorized into three clinical stages: early, intermediate, and late-stage AMD [14]. Early stage AMD is characterized by the presence of medium-sized drusen (>63 and ≤125 µm) without any impairment of visual function. Intermediate-stage AMD is defined as the presence of a large drusen (>125 µm) and/or abnormalities in the RPE. Late-stage AMD (advanced AMD) is classified into two clinical forms: the dry or nonexudative form (GA) and wet or exudative form (neovascular AMD). GA is defined as the irreversible loss of RPE and photoreceptor cells with progressive vision loss. Neovascular AMD is characterized by the invasion of newly immature choroidal blood vessels that break through the BM into the retina, causing exudates or hemorrhages (Figure 2).

## 3. Epidemiology and Risk Factors of AMD

Both environmental and genetic factors can contribute to the development and progression of AMD [15]. The observation of 52 common and rare variants at 34 genetic loci, independently associated with late AMD, implies that genetic factors might represent a crucial aspect in the management of the disease. A strong genetic association in AMD pathogenesis was found for complement factor H (CFH) [16].

The CFH gene was initially identified first on chromosome 1q31 as relevant to AMD [17]. The next strong risk locus identified through genome-wide association studies (GWASs) was reported a year later on 10q26 chromosomes [18], which turned out to be associated with age-related maculopathy susceptibility 2 (ARMS2), and high-temperature requirement A serine peptidase 1 (HTRA1) genes [19]. Polymorphisms in the CFH and ARMS2/HTRA1 genes clearly indicate that genetic predisposition is the key factor in the etiology of AMD [20].

Although polymorphisms in the CFH and ARMS2/HTRA1 genes allow for the highest single attributable risk for a single allele, other genetic variants have also been related to AMD. Notably, these are near genes of the complement system (CFB, CFI, C2, C3), Tissue Inhibitor of Metalloproteinases 3 (TIMP3), and Apolipoprotein E (APOE) [21]. Essentially, the discovery of rare genetic variants indicated the potential role of local inflammation and complement regulation in AMD pathogenesis. Gene–environment interactions and factors such as age and race may lead to oxidative damage and inflammation [22].

Despite intensive basic and clinical research, the pathogenesis of AMD has not been fully elucidated, probably due to its multifactorial nature. Epidemiologic studies have identified the key risk factors for AMD, with advanced age acknowledged as the main one and cigarette smoking coming in second [1,23,24]. Smoking is the important modifiable risk factor and showed a risk ratio of 2.46 for current smokers compared to nonsmokers [25]. Further modifiable risk factors are a high body mass index (BMI) and fat intake [26,27]. However, there is a continuing controversy about other modifiable factors such as lipid levels, blood pressure, light exposure, or alcohol intake, which are associated with a considerably greater risk of AMD [28,29].

## 4. Pathogenesis of AMD and Inflammation

The pathogenesis of AMD remains poorly understood. It is a multifactorial condition and is generally thought to be generated by intrinsic and extrinsic factors of the poorly degenerative RPE. Other causes include oxidative stress from the high metabolic demand of photoreceptors. Environmental risk factors such as cigarette smoke and aging are also known to have an effect. The accumulative damage caused by these combined factors presents as drusen [30].

Oxidative stress, reactive oxygen species, and lipid peroxidation are strongly associated with the risk factors of AMD. Retinal damage due to reactive radicals or light-induced singlet oxygen leads to the formation of lipid peroxides. Photoreceptors degenerate when exposed to a continuous oxidative challenge or when antioxidative defense mechanisms are impaired. Currently, the strongest evidence for a correlation between oxidative stress and AMD comes from AREDS [29,31]. They evaluated the use of high-dose vitamins C and E, beta-carotene, zinc, and copper in AMD. They found that patients with intermediate AMD or significant vision loss due to AMD in the second eye showed the greatest risk reduction for progression to advanced AMD and a three-line decrease in visual acuity. 

In healthy eyes, BM transports the oxygen and nutrients from the choriocapillaris to the RPE or transports the metabolic substance of the RPE to choriocapillaris [32,33]. BM thickening and formation of the drusen compromise the transport function of the BM, which consequently initiates BM degeneration or at least contribute to AMD [34,35]. Altered BM permeability impairs progressive RPE dysfunction [36]. As a result, substances that are usually removed by the choriocapillaris accumulate between the RPE and BM, resulting in the emergence of drusen [37]. Drusen are characteristic changes in early AMD and these deposits exceed those related to the normal aging process [38,39]. The appearance of drusen is associated with the thickening of the collagenous layers of BM, which is in turn associated with lipid and protein accumulation and increased glycation end products. These changes, which are the result of metabolic dysfunction, may contribute to AMD progression by compromising the passage of nutrients between the choroid and outer retina and by creating a hypoxic environment, the consequence of chronic inflammatory damage to the choriocapillaris.

The importance of inflammation in AMD has remarkably increased in recent years. Evidence for the role of chronic inflammation in AMD was found not only in the retina and in the blood, but also in drusen. Specifically, drusen were shown to contain complement activators, complement fragments, and a membrane attack complex (MAC) [40,41]. Hageman et al. suggested their inflammatory effects in AMD, proposing activation and recruitment of choroidal dendritic cells by locally injured RPE cells and subsequent drusen formation [42].

There is strong evidence that complement abnormalities play a central role in the pathogenesis of AMD, with mutations in the complement factor H gene that increase the risk of AMD by 2.7–7.4-fold [43,44]. Soft drusen contain C3a and C5a and induces the upregulation of vascular endothelial growth factor (VEGF) in RPE, which is consistent with the increased risk of CNV associated with soft drusen [45]. Additionally, various complement factors and MAC are localized in drusen and in compromised RPE cells of AMD eyes [46,47]. The association of genetic risk was also found in other complement genes; as such, the complement system and its components were studied as therapeutic targets [48,49,50]. 

## 5. Complement Cascade

**Figure 3:**
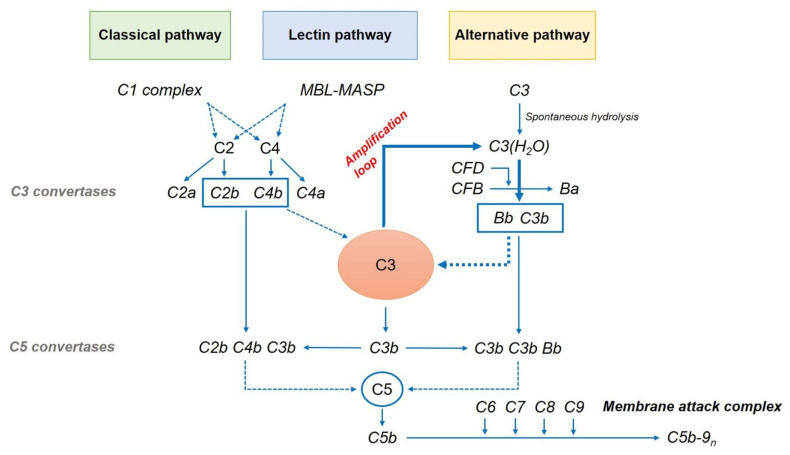
The complement cascade. MBL, mannose-binding lectin; MASP, MBL-associated serine protease; CFB, complement factor B; CFD, complement factor D.

Drusen and lipofuscin-related products contain several pro-inflammatory factors, including complement pathway components, that have been identified as primary contributors to AMD development [51]. The complement system is an integral part of the innate immune response that defends the host against foreign pathogens and modifies antigen-specific immune and inflammatory responses. It comprises three pathways: classical, lectin-dependent, and alternative pathways (Figure 3). Each pathway has its own activation mechanism, although they seem to combine sometimes to stimulate the same protein—that is, complement component 3 (C3). The cascade activation of these pathways leads to the synthesis of the MAC. It can form a channel that penetrates the cell membrane, leading to cell death.

### 5.1. Classical Pathway

The classical pathway is usually related to antigen–antibody complexes. It is initiated by binding between C1q and antibodies. After the activation of the C1 complex, it divides the complement C2 and C4 into C2a, C2b, C4a, and C4b. C4b and C2b bind to C4b2b, which is a C3 convertase. C3 convertase catalyzes the proteolytic cleavage of C3 into C3b and further binds to C3b to form a C5 convertase.

### 5.2. Lectin Pathway

The lectin pathway is initiated by the interaction of mannose-binding lectin (MBL) with mannose-containing polysaccharides found on microorganism surfaces. This initiates the cleavage of C4 and C2 by the MBL-associated serine protease complex. The C4b2b complex forms a C3 convertase of the lectin pathway [52].

### 5.3. Alternative Pathway

An alternative pathway is closely implicated in the pathogenesis of AMD. This is related to the spontaneous hydrolysis of a thioester bond in C3 to form C3(H_2_O), which further binds to complement factor B (CFB). This conformational change allows Factor D, a constitutively active serum protease, to cleave FB into Ba and Bb. Factor Bb is retained within the C3(H_2_O)–Bb complex, where it acts as a serum protease for C3, cleaving it to C3b. This can in turn associate with FB to generate more C3 convertase (C3bBb). This auto-activation process is known as “tickover.” The resulting C3 convertase initiates the terminal pathway as an “amplification loop” of the complement cascade, generating more C3b and C3a from C3. It plays an important role in producing the final product membrane attack complex (MAC) and cell destruction [53,54].

### 5.4. Common Variations in Complement Proteins, CFH

The continuous control of the alternative pathway is necessary due to the spontaneous nature of the activation, its amplifying properties, and its potential to stimulate cell lysis. The major negative regulator of the alternative pathway is CFH. It is a protein composed of 20 domains known as short consensus repeats (SCRs) or complement control protein modules [55,56]. CFH is locally produced by RPE and accelerates to C3 convertase decay, preventing the amplification of C3b deposition.

Significant improvements in sequencing technologies and GWAS have offered insights into the genetics of AMD phenotypes. In 2005, several studies on genetic association proved that the CFH gene on chromosome 1q32 is the first gene associated with AMD risk [57,58]. The rs1061170 polymorphism, which leads to an amino acid change at position 402 of the CFH protein (Y402H), was recognized to have the clearest association with AMD risk. Although the prevalence of the 402H risk variant differs across ethnicities, the presence of at least one CFH risk allele is estimated to account for a population attributable risk fraction for early and late AMD of 10% and 53%, respectively [59,60,61].

A more recent meta-analysis stratified by stage of disease and ethnicity supported that the polymorphism is significantly associated with AMD. The analysis found that the mutated allele showed a 1.44, 2.90, and 2.46 risks of early AMD, dry AMD, and wet AMD, respectively [62]. In contrast, the rs800292 polymorphism as a coding variant in the SCR1 domain has been found to be protective against AMD in both Caucasians and Asians. It also showed a better treatment response to wet AMD [63]. These results demonstrate that various CFH genetic variants are significantly associated with the risk of AMD and that further research is needed.

### 5.5. Other Common Variations in Complement Proteins, C3, CFB, and CFI

As previously stated, a meta-analysis of GWAS identified 19 SNPs, of which four were contained within complement cascade genes (CFH, CFI, C3, C2/CFB) [64,65]. In addition, these rare coding variants have also been identified [66].

#### 5.5.1. C3

C3 protein is in a biologically inactive state until it exposes binding sites for the pathogenic cell surface and other complement components [67]. A variant polymorphism in C3 (rs2230199) is the most commonly investigated polymorphism and is associated with AMD risk [68]. In contrast, the rs2250656 polymorphism has been found to be protective against AMD [69]. The role of C3 genetic variants in response to AMD treatment is still controversial [70,71].

#### 5.5.2. Complement Factor B (CFB) and C2

Polymorphisms in the CFB gene, the common rs641153 polymorphism (R32Q), are associated with a lower AMD risk [72]. C2 is a serum glycoprotein related to the classical pathway of the complement system. Two polymorphisms, namely, rs9332739 and rs547154, are known to lower the AMD risk by 45% and 53%, respectively [73]. The rs9332739 and rs547154 polymorphisms in C2 are noncoding variants, whereas the rs641153 polymorphism in CFB results in reduced alternative pathway amplification and hemolytic activity of the CFB protein [74,75]. However, evidence on the effect of CFB and C2 genetic variants on the response to AMD treatment is also still lacking [76].

#### 5.5.3. CFI

The CFI gene is encoded as a precursor protein in macrophages, endothelial cells, lymphocytes, and fibroblasts. To convert the active protein, the precursor is cleaved to form a heterodimeric glycoprotein. This can prevent the assembly of convertase enzymes by cleaving C4b and C3b. Fagerness et al. first demonstrated the association between CFI polymorphisms and AMD in 2009 [48]. The rs10033900 polymorphism as the most investigated polymorphism is confirmed to lower the risk of AMD in Caucasians [77].

## 6. Experimental Evidence for Complement Cascade Dysfunction in AMD

Complement activation is well known to be associated with AMD, and animal models have provided data to disrupt the complement pathway and AMD pathogenesis. Various models have provided an understanding of the complement in several AMD-associated progression mechanisms. Early studies focused on the effects of complement in murine models of wet AMD, which are traditionally made by laser-induced CNV [78]. It is a commonly used experimental method that induces breaks in BM and triggers neovascularization from the choriocapillaris. However, it most likely reflects a wound healing response and not disease progression [79]. Despite this limitation, this method has been widely used to determine the role of complements in CNV development and progression with the alternative pathway [80,81]. Experimental studies have demonstrated that the inhibition of complement activation via systemic or local pathways can suppress laser-induced CNV. Animal models have shown that the inhibition of C3a, C5a, CFB, and MAC or the administration of the complement regulatory molecules CD59 and CFH can suppress the development of CNV [82,83,84]. Excessive activation of the alternative pathway increased the CNV size in CFH-deficient mice [85]. In addition, Rohrer et al. reported that mice lacking the alternative pathway (CFB-deficient) showed lower CNV than did those lacking other complement pathways [84,86]. Moreover, it was reported that RPE could express some complement proteins [87,88], and complement attack could damage the RPE cells [89,90]. Inhibition of the complement pathway in the RPE rescued the photoreceptors in a mouse model of Stargardt macular degeneration [91]. These reports clearly demonstrated that the complement system was involved in RPE damage, finally leading to photoreceptor degeneration. Nevertheless, the specific mechanism of the complement pathways on CNV development and RPE damage in AMD remains unclear.

## 7. Potential Treatment Targeting Specific Complement Components in AMD

The association of VEGF with the pathogenesis of neovascular AMD and the introduction of anti-VEGF therapy as the gold standard treatment has significantly altered its prognosis. However, it has been focused mainly on delaying and reversing vision loss caused by late stage of the disease, known as exudative AMD. 

Although no drugs are currently available for the treatment of dry AMD, several therapeutic trials are at differing levels of clinical development. The drugs in these trials target complement components related to the alternative complement pathway (Table 2). Complement 3 is a striking target in the complement pathway, considering its role in AMD development. However, its powerful effect makes it impossible for us to completely disregard the risk of inhibiting its function. The complement system plays an important function as the first defense line of the innate response, protecting the human organism by recognizing and mediating the removal of pathogens, debris, and dead cells. The balance between its therapeutic benefits and risks brought out by general inhibition should be distinguished.

### 7.1. C3

#### 7.1.1. POT-4 (AL-78898A)

POT-4 (AL-78898A) is a compstatin analog that selectively binds to C3b and C3c [92,93]. It is the first complement inhibitor to be tested in AMD, and previous experiments on eight monkeys showed encouraging results [94]. Diffusion of drusen in the macula was found in four monkeys after 6 months, and partial disappearance of drusen was further observed after 9 months in all monkeys. These favorable results were also shown in a Phase 1 trial (NCT00473928) [95]. However, phase 2 trials of POT-4/AL-78898A were terminated before the primary endpoint was met, and it was impossible to verify the data of their final efficacy (NCT01603043) [96]. Despite this, AL-78898A remains the first valuable complement inhibitor drug to be evaluated and more trials are needed.

#### 7.1.2. APL-2

APL-2 is a modified form of POT-4 that has a longer half-life. In its Phase 2 trial (FIL-LY) [97], GA lesion growth was lower by 29% in the APL-2 treatment group than that in the control group after 12 months of monthly intravitreal injection. At post hoc evaluation in the second 6 months of the trial, the monthly treatment group showed a significant, 47%, reduction in lesion growth, while the every-other month treatment group showed a 33% reduction. This suggests that a higher frequency of injection is associated with a better outcome. However, it is also notable that APL-2 therapy was associated with a higher risk of developing wet AMD, with the risk increasing with the frequency of injection. While the control group only had a 1% risk of AMD, the every-other month treatment group had an 8% risk, and the monthly treatment group had an even higher risk of wet AMD at 18%. However, it remains unclear whether there is any relation between APL-2 and wet AMD, and this issue should be taken seriously. A phase 3 clinical trial for GA is currently recruiting participants (NCT03525613). Furthermore, a phase 2 clinical trial for patients with wet AMD is also in progress (NCT03465709).

### 7.2. C5

#### 7.2.1. Eculizumab

Eculizumab is a humanized monoclonal antibody targeting the complement protein C5 and specifically binds to it. It inhibits cleavage to C5a and C5b during complement activation, ultimately preventing MAC formation. It is approved by the United States Food and Drug Administration (FDA) and European Medicines Agency (EMA) for the treatment of other genetic deficiencies of complement inhibition, such as paroxysmal nocturnal hemoglobinuria and atypical hemolytic uremic syndrome [98].

The Phase 2 COMPLETE trial (NCT00935883) [99] evaluated eculizumab in 30 patients with GA. The patients received either high-dose eculizumab (900 mg for 4 weeks followed by 1200 mg every 2 weeks) or low-dose eculizumab (600 mg for 4 weeks, followed by 900 mg every 2 weeks) until week 24. The other ten patients in the placebo group were observed over 24 weeks [96]. The primary efficacy endpoint was the change in the GA area at 26 weeks. However, GA enlargement did not significantly differ between the eculizumab treatment and placebo groups at 26 weeks (0.19 ± 0.12 vs. 0.18 ± 0.15 mm, respectively, *p* = 0.96). At 52 weeks, the GA area also increased in both groups (0.37 ± 0.22 mm and 0.37 ± 0.21 mm, respectively), with no significant difference (*p* = 0.93). No drug-related adverse events were reported, but this trial also showed no beneficial effect of eculizumab treatment in reducing drusen volume [100,101].

There are several issues regarding the intravenous use of GA drugs. The rationale for using a systemic drug is based on evidence that complement activation in the choroid plays an important role in the progression of GA. Although systemic complement inhibitors are successfully used for other systemic diseases, in GA, the amount of drug delivered systemically could be insufficient to penetrate the blood retinal barrier and affect GA progression [102].

#### 7.2.2. LFG316

LFG316 is a human monoclonal C5 antibody. In a phase 2 clinical trial (NCT01527500) of 150 patients with GA [103], there was no significant difference in lesion size between the intravitreal LFG316 treatment group (5 mg/50 μL) and the sham group during a 12-month follow-up. A phase 2 clinical trial (NCT01624636) with intravenous LFG316 injection was also terminated early [104].

#### 7.2.3. Zimura (ARC1905)

Zimura (ARC1905) is a selective C5 inhibitor that was initially used in combination with anti-VEGF agents to achieve more therapeutic benefits than those with anti-VEGF monotherapy. Combined anti-VEGF therapy with a complement inhibitor such as Zimura is believed to have the potential to increase the efficacy of anti-VEGF monotherapy in wet AMD. The phase 2 NCT03362190 trial [105] aims to establish the safety and tolerability of Zimura with Lucentis^®^ (0.5 mg) in wet AMD, but the final results are yet to be reported. Another phase 2 trial (NCT02686658) [106] for the safety and efficacy of intravitreous Zimura monotherapy in GA patients has been concluded, and the data are currently being analyzed.

### 7.3. Complement Factor D (CFD)

#### Lampalizumab (FCFD4514S)

Lampalizumab (FCFD4514S/anti-factor D) is an intravitreally administered antigen-binding fragment (Fab) of a humanized monoclonal antibody targeting complement factor D (CFD). It binds to CFD and prevents CFD-mediated activation of C3bBb and effectively terminates the alternative complement pathway [107]. Preclinical tests in monkeys have shown that intravitreal clinically relevant doses have minimal systemic inhibitory effects against the alternative complement pathway [108,109].

The MAHALO Phase 2 study (NCT01229215) assessed lampalizumab in patients with GA. However, although the treatment group showed a 20% reduction in GA progression, there was no significant difference compared to the sham groups [110]. The phase 3 trials CHROMA (NCT02247479) and SPECTRI (NCT02247531) were designed as double-masked, multicenter, randomized, sham injection-controlled trials [111,112]. These studies enrolled a total of 936 patients and the largest GA complement. The patients were randomized in a 2:1:2:1 ratio to receive 10 mg lampalizumab and sham injection every 4 weeks and lampalizumab and sham injection every 6 weeks. The primary efficacy endpoint was the mean change in the GA area at 12 months. However, the results showed that despite 48 weeks of treatment, lampalizumab did not reduce GA enlargement. None of these changes were statistically significant.

## 8. Conclusions

**Figure 4:**
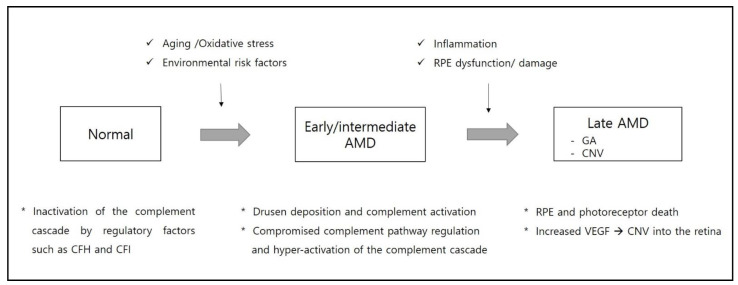
Schematic representation of the pathogenesis from early to late stages of age-related macular degeneration (AMD). CFH, complement factor H; CFI, complement factor I; CNV, choroidal neovascularization; GA, geographic atrophy; RPE, retinal pigmented epithelium; VEGF, vascular endothelial growth factor.

AMD is a complex multifactorial disease which leads to irreversible retinal damage and vision loss. The complement cascade is known to play an important role in pathogenesis of AMD. Additionally, the discovery of genetic variants in genes for complement proteins highlights the role of chronic inflammation and complement regulation in AMD pathogenesis (Figure 4).

Considering the probable role of the complement system in the development and progression of AMD, many clinical trials investigating the effect of complement inhibitors have been conducted or are in progress. The results of clinical trials have shown that only a subgroup of patients responded favorably. Although several phases 2 and 3 clinical trials are already in progress, there is still no encouraging clinical trial evidence to support this hypothesis.

However, experimental evidence suggests that the complement system is a promising target for the development of novel therapies, which could support conventional treatment. Further research about the pathogenesis of AMD linked with the complement system will expand AMD’s pathophysiology, and it will contribute to identifying additional potential therapeutic targets.

## Figures and Tables

**Table 1:** The Beckman clinical classification of age-related macular degeneration (AMD).

AMD Classification	Definition
No AMD	No drusen and no AMD pigmentary changes
Normal aging changes	Small drusen (≤63 µm) and no AMD pigmentary abnormalities *
Early AMD	Medium sized drusen (>63 µm and ≤125 µm) and no pigmentary changes
Intermediate AMD	Large sized drusen (>125 µm) and/or pigmentary changes
Late AMD	Neovascular AMD or geographic atrophy

* AMD pigmentary abnormalities represent any definite hyperpigmentary or hypopigmentary abnormalities associated with medium or large drusen but not associated with known disease entities. 125 µm is approximately as wide as a major branched retinal venule crossing the optic disc margin, and the size indicates the diameter of drusen.

**Table 2:** Noteworthy complement therapeutics in clinical trials.

Drugs	Targets	Drugs Administration	Status	Primary Outcomes	ClinicalTrial.Gov
POT-4, AL-78898A	C3; peptide inhibitor	Monthly intravitreal	Phase II completed	Safety; mean reduction in central subfield retinal thickness	NCT00473928 NCT01157065
APL-2	C3; peptide inhibitor	Monthly intravitreal	Phase III recruiting	Changes in GA area measured by FAF	NCT03525600 NCT03525613
Eculizumab	C5; monoclonal antibody	Intravenous	Phase II completed	Growth of GA and decrease in drusen volume	NCT00935883
LFG316	C5; monoclonal antibody	Monthly intravitreal	Phase II completed	GA lesion growth measured by FAF	NCT02515942
Zimura (ARC1905)	C5; aptamer	Monthly intravitreal	Phase III recruiting	Safety; mean rate of change in GA measured by FAF	NCT04435366(Dry) NCT03362190(Wet)
Lampalizumab (FCFD4514S)	Complement factor D; monoclonal antigen-binding fragment (Fab)	Monthly intravitreal	Phase III completed	Changes in GA area measured by FAF at 1 year	NCT02247479 NCT0224753

GA, geographic atrophy; FAF, fundus autofluorescence.

## Data Availability

All relevant data are included within the manuscript.

## Abbreviations

AMD	age-related macular degeneration
AREDS	age-related eye disease study
BM	Bruch’s membrane
CFB	complement factor B
CFD	complement factor D
CFH	complement factor H
CFI	complement I
CNV	choroidal neovascularization
GA	geographic atrophy
MAC	membrane attack complex
MBL	mannose-binding lectin
RPE	retinal pigment epithelium
VEGF	vascular endothelial growth factor
